# The Development of New Functional Foods and Ingredients

**DOI:** 10.3390/foods13193038

**Published:** 2024-09-25

**Authors:** Jeung Hee Lee, Mi Jeong Kim, Choon Young Kim

**Affiliations:** 1 Department of Food and Nutrition, Daegu University, Gyeongsan 38453, Republic of Korea; jeunghlee@daegu.ac.kr; 2 Interdisciplinary Program in Senior Human Ecology, Changwon National University, Changwon 51140, Republic of Korea; mjkim@changwon.ac.kr; 3 Department of Food and Nutrition, Changwon National University, Changwon 51140, Republic of Korea; 4 Research Institute of Human Ecology, Yeungnam University, Gyeongsan 38541, Republic of Korea; 5 Department of Food and Nutrition, Yeungnam University, Gyeongsan 38541, Republic of Korea

Functional foods have emerged as a crucial area of research and innovation in the realm of food science, offering promising solutions for the prevention and management of chronic diseases such as cardiovascular disease, cancer, osteoporosis, diabetes, and hypertension. These foods, enriched with bioactive nutraceutical compounds, present nutritional and therapeutic benefits. The synergy between the technological and physiological properties of functional ingredients has paved the way for improvements in food quality, such as enhanced organoleptic properties and increased stability against oxidation and emulsification. These advancements support the development of novel functional food products that align with recent manufacturing trends and consumer preferences.

This Special Issue of *Foods* brings together the latest research and developments in the field of functional foods and nutraceuticals. From exploring innovative bioactive nutraceuticals to investigating the mechanisms underlying their health benefits, this collection of research highlights the dynamic and interdisciplinary nature of functional food science. The contributions also delve into cutting-edge technologies for food processing and the development of plant-based functional foods.

The aim of this Special Issue is to provide a comprehensive overview of the current trends and future directions in functional food research. Covering a diverse range of topics, the Issue presents novel insights into the development of functional foods and their applications. Each article contributes to the understanding of how these ingredients can be developed and applied in creating sustainable and high-value-added food products that prevent chronic diseases and promote overall health. The following sections summarize the individual research articles, categorized by their contribution to these themes.

(1) The research in this Special Issue explored the therapeutical potential of bioactive compounds in functional foods. Rarison et al. examined the biological properties of lipophilic extracts from *Polyscias fruticosa*, a plant used in traditional medicine [contribution 1]. The investigation employed network pharmacology, in silico, and in vitro methodologies to identify 71 compounds exhibiting antioxidant and anti-inflammatory properties.
(2) Key compounds like phytosterols and sesquiterpenes regulate genes involved in oxidative stress and inflammation, offering protective effects in a RAW264.7 macrophage cell model. These findings highlight the potential for developing nutraceuticals from the plant’s lipophilic extracts. Another study explored the anti-inflammatory properties of the methanolic extract from the aerial parts of *Heracleum moellendorffii* Hance (HmAPE) [contribution 2]. The extract was found to significantly reduce nitric oxide production and downregulate the expression of inflammatory markers in lipopolysaccharide-treated macrophage cells. This effect was mediated through the activation of the Nrf2/HO-1 pathway. These findings suggest potential anti-inflammatory applications for HmAPE in the development of functional foods.
(3) Several researchers examined the development of functional foods with plant-based ingredients. Navarro-Cortez et al. aimed to evaluate the phenolic and flavonoid content, along with the antioxidant activity, in mixtures of Mexican plants including *Portulaca oleracea*, *Chenopodium album*, *Opuntia oligacantha*, and *Amaranthus tricolor* [contribution 3]. The results showed that the binary mixture of *Portulaca* and *Chenopodium* had the highest phenolic content and antioxidant potential, suggesting their potential application in functional foods to enhance antioxidant intake. Alfheeaid et al. developed high-energy high-protein bars using either Sukkari dates or a dried fruit mixture and compared their composition [contribution 4]. A date-based bar (DBB) had higher ash, crude fiber, and certain minerals than a fruit-based bar (FBB), while the FBB contained more moisture, fat, and different sugars. Both bars had similar protein content and calorie values. The DBB excelled in most terms of the essential amino acids and specific minerals, whereas the FBB had higher values of phytochemicals and antioxidant activity. The sensory tests showed a preference for the DBB. The research recommends using Sukkari dates in commercial protein bars due to their nutrient density and economic benefits.
(4) Two studies in the Special Issue explored novel technology and processes. Jeong et al. addressed novel technology and processes in the formulation and production of functional foods [contribution 5]. This study focused on optimizing the processing conditions for creating gluten-free high-protein laver chips using air-frying and reaction flavor technologies. The research utilized response surface methodology to determine the ideal composition of ingredients such as dried laver, surimi, and rice flour. The chips were subjected to air-frying, resulting in a product with desirable sensory characteristics and an elevated puffing ratio. Various flavor-inducing solutions were incorporated to improve the sensory attributes of the chip while maintaining a low-calorie content. This innovative approach focused on creating a nutritious snack with improved texture and flavor, addressing modern dietary needs for high-protein, low-calorie, and gluten-free options. Another study presented the innovative use of electroencephalography (EEG) to analyze brain function after consuming germinated wheat beverages. Aung et al. investigated the impact of hot germinated wheat beverages (HB) and cold germinated wheat beverages (CB) on brain activity using EEG [contribution 6]. They found that drinking the hot beverage significantly increased delta and theta brain waves, which are associated with relaxation and calmness. In contrast, consuming the cold beverage led to a decrease in alpha and beta waves, which are linked to alertness and concentration. The topographic analysis also showed that the hot beverage had a more pronounced effect on brainwave activity than the cold one. These findings suggest that the temperature of the beverage influences the cognitive states, with HB promoting relaxation and CB enhancing alertness, making this study valuable for understanding how beverage temperature can affect cognitive function.

As the field of functional foods continues to evolve, it is essential to explore more sustainable and innovative approaches to nutraceutical development and food processing. The Special Issue emphasizes not only the therapeutic potential of functional ingredients but also their technological advantages for food manufacturers aiming to meet the growing demand for healthier food products. We believe this Special Issue will inspire future research that bridges the gap between food technology and health outcomes. By leveraging new bioactive compounds and delivery systems, we can further improve the nutritional and therapeutic efficacy of foods, contributing to the global fight against chronic diseases.

## Data Availability

Not applicable.

